# 397. Hospitalizations Associated with Disseminated Coccidioidomycosis and Coccidioidal meningitis, Texas, 2016-2023, USA

**DOI:** 10.1093/ofid/ofaf695.135

**Published:** 2026-01-11

**Authors:** Chun Ho Szeto, Alfredo Chavez Morales, John Garza, Fariba Donovan

**Affiliations:** Texas Tech University Health Sciences Center at Permian Basin, Odessa, TX; Texas Tech University Health Sciences Center at Permian Basin, Odessa, TX; Texas Tech University Health Sciences Center at Permian Basin, Odessa, TX; University of Arizona College of Medicine-Tucson, Tucson, Arizona

## Abstract

**Background:**

Coccidioidomycosis is a fungal infection caused by inhalation of dimorphic fungi *Coccidioides* species spores [1]. Approximately 1% of infections progress to disseminated coccidioidomycosis (DC), with coccidioidal meningitis (CM) representing its most severe form [2]. Epidemiological data on DC in Texas remain limited. This study aims to characterize the demographic characteristics and geographic distribution of DC hospitalizations in Texas.

**Figure:**
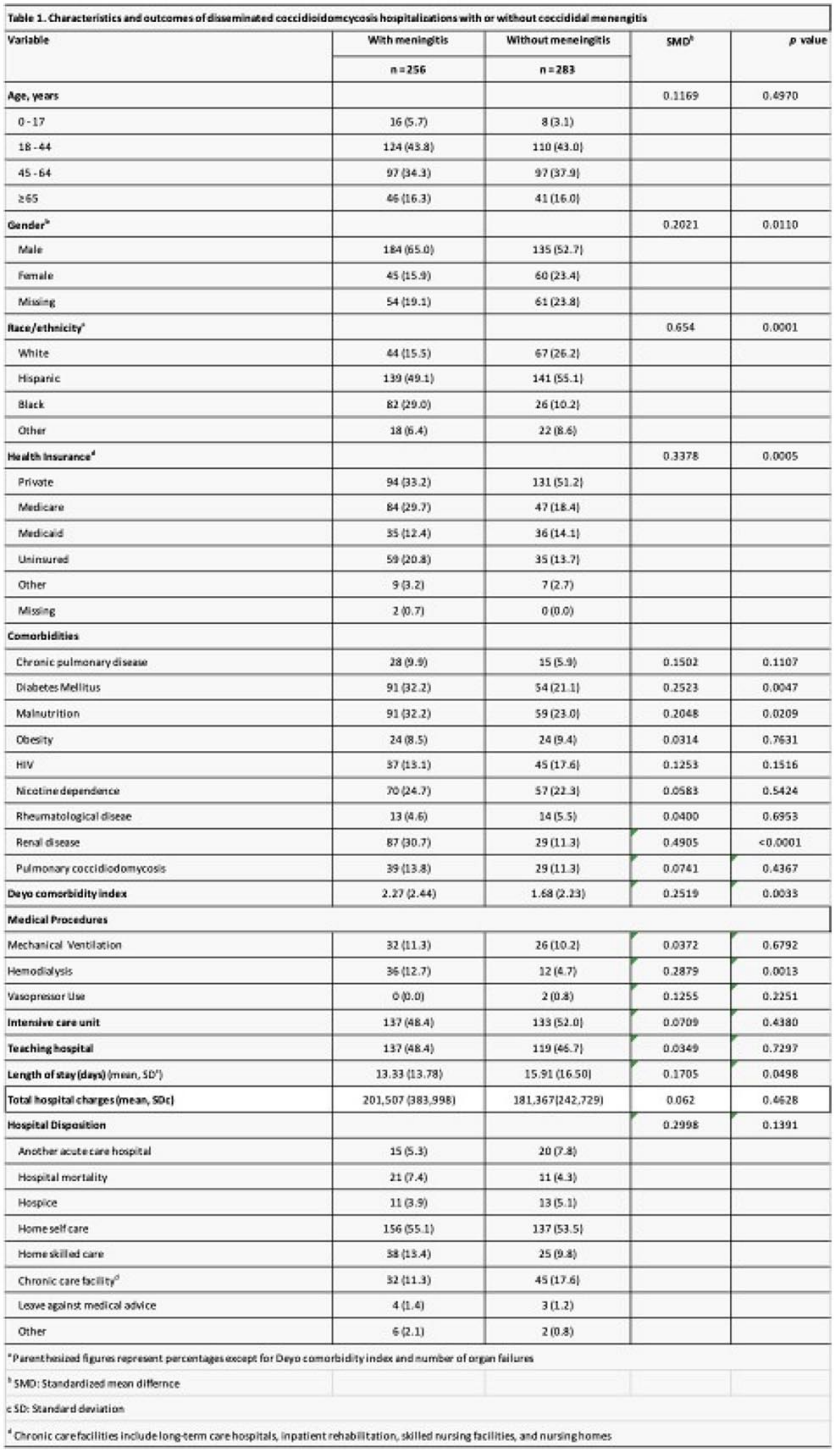

Counties in Texas where hospitalized patients with disseminated coccidioidomycosis resided, 2016-2023

**Figure:**
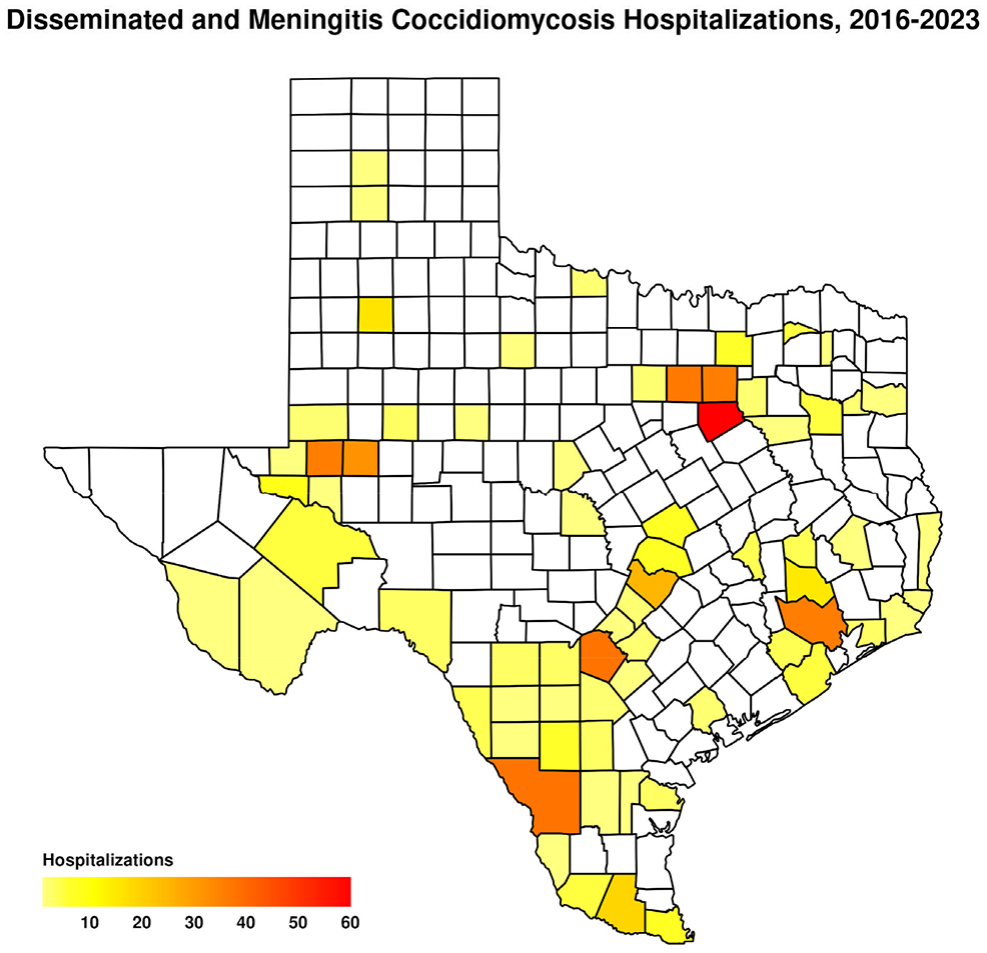

Odessa–San Angelo public health region had the highest proportion of DC hospitalizations (17.3%), followed by Dallas–Fort Worth (16.4%).

**Methods:**

We analyzed inpatient public-use data files from January 1, 2016, to December 31, 2023, obtained from the Texas Health Care Information Collection, Texas Department of State Health Services, Center for Health Statistics. Hospitalizations with a diagnosis of DC were identified using ICD-10-CM codes B38.4 and B38.7. The only exclusion criterion was residency outside of Texas. The DC cohort was stratified into two groups: (1) DC with CM and (2) DC without CM. Patients' residential regions were categorized and mapped according to public health regions.

Inclusion and exclusion critieria for study of disseminated coccidiodomycosis-related hospitalizations, Texas, USA, 2016-2023

**Figure:**
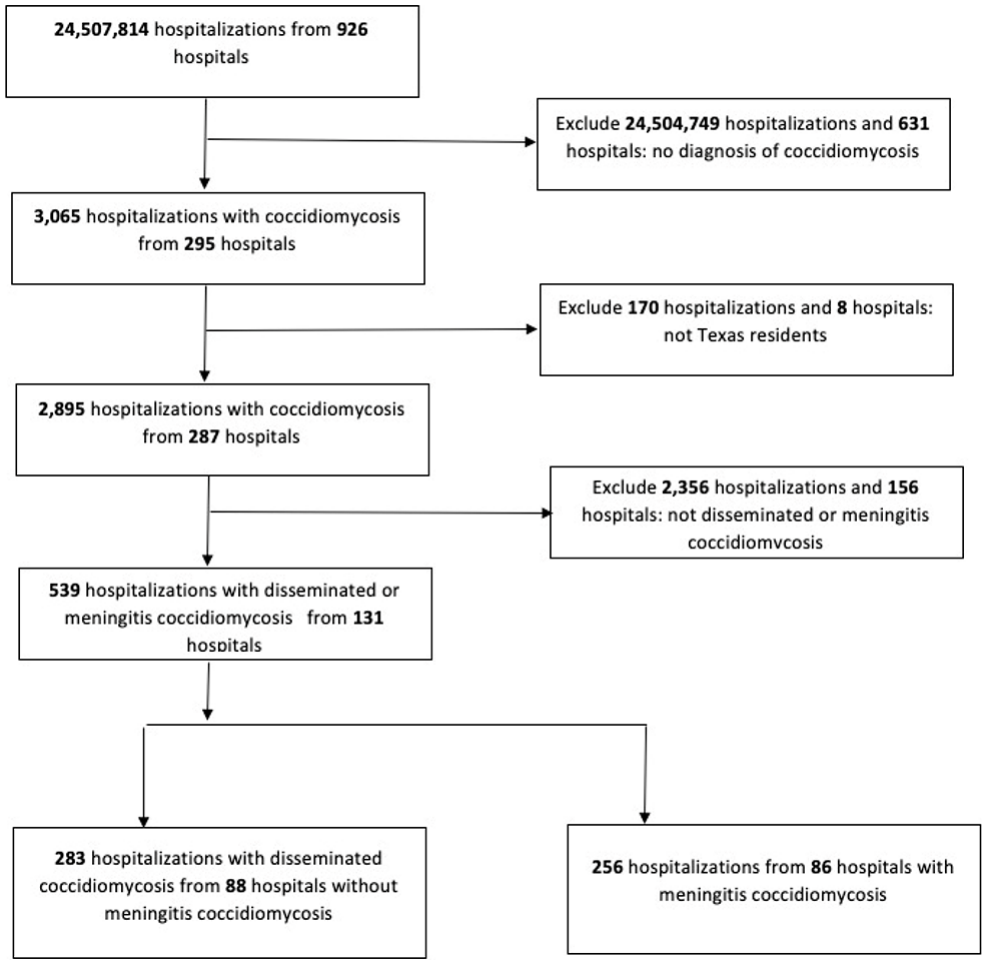

**Results:**

A total of 539 hospitalizations for DC were identified. Of these, 47.5% (256/539) had CM, and 52.5% (283/539) had DC without CM. Overall, 59.1% of patients were male. The majority were aged 18–44 years (43.4%), followed by those aged 45–64 years (36.0%) (Table 1). In both CM and non-CM cohorts, age 18-44 was the predominant group. Pulmonary coccidioidomycosis was documented in 12.6% of DC cases. The most common comorbidities were diabetes mellitus (26.9%) and nicotine dependence (23.5%). HIV infection was present in 15.2% of hospitalizations. Notably, 50.0% of cases required intensive care unit admission. The Odessa–San Angelo public health region had the highest proportion of DC hospitalizations (17.3%), followed by Dallas–Fort Worth (16.4%).

**Conclusion:**

Our findings highlight a substantial burden of DC in Texas, particularly in West Texas. Unexpectedly, high hospitalization rates were also observed in metropolitan regions not previously considered endemic. These results underscore the evolving geographic distribution of coccidioidomycosis in Texas and support the need for increased epidemiological surveillance. Early recognition and risk stratification are essential to prevent severe complications such as CM.

**Disclosures:**

All Authors: No reported disclosures

